# Food price perception, food and beverage marketing and the nutritional status of children 6–24 months in Obunga slums, Kisumu Kenya: a cross-sectional study

**DOI:** 10.1186/s40795-023-00772-3

**Published:** 2023-10-06

**Authors:** Umotho Kinya Mbae-Mugambi, Agatha Christine Onyango, David Omondi Okeyo

**Affiliations:** 1 School of Public Health & Community Development, Maseno University, Kisumu City, Kenya; 2 Kenya Nutritionists and Dieticians Institute, Nairobi City, Kenya

**Keywords:** Food price perception, Food and beverage marketing, Nutritional status

## Abstract

**Background:**

Malnutrition is a significant problem in Africa. In Kenya, 26% of under-fives are stunted; slums are the hardest hit. Obunga slum has the highest prevalence at 40%.

**Methods:**

A cross-sectional study was adopted; simple random sampling techniques were used to identify 189 eligible households in the Obunga slum with children between 6–24 months. An interviewer-administered questionnaire collected data on food price perceptions and food and beverage marketing. An anthropometric data collection form gathered information on the children’s height, weight and age. Scores for stunting, wasting and underweight were generated based on WHO Z-Score cut-off points. Binary logistic regression identified the relationship between food price perceptions, food and beverage marketing and the nutritional status of children between 6–24 months in Obunga slums.

**Results:**

Prevalence of wasting was 3.2%, stunting was 27.0%, underweight was at 7.4%, while overweight was at 13%. *Food price perceptions:* An increase in fruits prices was significantly associated with wasting (Adjusted O.R. = 10. 82, C.I. = 1.10—106.77, *P* < 0.05) and underweight (Adjusted O.R. = 5.44, C.I. = 1.35—21.61, *P* < 0.05). *Food & Beverage Marketing:* Feeding children on commercially produced complementary food products and commercially produced food was significantly associated with wasting at an (Adjusted O.R. = 7.82, C.I. = 1.29—47.46, *p* < 0.05, and adjusted O.R. = 5.96, C.I. = 1.06—33.60, *p* < 0.05) respectively. Stunting was significantly associated with listening/reading or watching advertisements on commercial food products (Crude O.R. = 0.49, C.I. = 0.24—0.998, *p* < 0.05.). Watching food-related adverts on television (Adjusted O.R. = 0.38 C.I. = 0.146- 0.10) and watching marketing on commercial foods (Adjusted O.R. = 0.21, C.I. = 0.07—0.61) and watching television (Adjusted O.R. = 9.30, C.I. = 2.31—37.40). While watching food-related adverts on television was associated with being underweight (Adjusted O.R. = 18.68 and at C.I. = 1.22—286.89).

**Conclusion:**

The price perceptions of fruits, feeding children commercially produced food products and complementary foods, and Watching food-related adverts on television; had an impact on the nutritional status of children. Thus, a longitudinal study would be needed to understand the long-term effect of food prices and food and beverage marketing on nutritional status.

**Supplementary Information:**

The online version contains supplementary material available at 10.1186/s40795-023-00772-3.

## Introduction

Malnutrition remains a health challenge in low and middle-income countries globally. Undernutrition remains the biggest challenge, reflected by the increasing prevalence of stunting, wasting and being underweight. Stunting was at 22.3% globally, while in Africa, stunting is currently at 30.7% [1, 2]. As of 2019, in Kenya, the prevalence was lower at 4% wasting, 11% underweight and stunting at 26% [3]. The newly released health demographic survey reveals that stunting in the country has reduced to 18% [4]. However, data on the urban slums is still not reflected in the survey. The Kenyan urban slums tell a different story, with the prevalence of stunting, an indicator of chronic malnutrition, reaching over 35% [5, 6].

Obunga slums in Kisumu had the highest prevalence of stunting at 40.2% [7], despite efforts being put in place regarding intervention programs targeting mitigating the increasing prevalence of malnutrition. This article focuses on children between 6–24 months, representing the largest majority of stunted children under five years [6, 8, 9]. However, after 24 months, stunting is irreversible [10, 11]. Focus has been given to a combination of socio-demographic factors, with little attention to food systems. This study was based on a conceptual framework model by Neff et al. 2009 [12], emphasizing the relationship between food systems and health disparities. This model provides a schematic relationship explaining that diet disparities directly influence health. Diet disparities are also influenced directly by broad food systems [12]. This study used the model to investigate the relationship between nutrition status and broad food systems characterized by food price perceptions and food and beverage marketing. Food price is an essential determinant in food purchases [12]. Rising food prices affect people in poorer countries the most [13]. A critical note is that the persons most vulnerable to food prices are those with few coping mechanisms and who spend a considerable proportion of their income on food purchases. They usually include pastoralists, people without land, and the urban poor [14]. According to Neff et al., 2009 [12], the inability to buy and afford adequate and nutritious foods usually exacerbates health disparities and has a multi-generational effect. Food and beverage marketing promotes particular brands and food categories [12]. Adolescents and children are targeted by intensive food & beverage marketing, as they influence household purchases through nag and pester power [15]. The heavy marketing toward the youth, especially children, hopes to build positive associations with brands, ensuring brand loyalty even as future adult consumers [15]. Food and beverage marketing has also been shown to influence complementary feeding. Mothers believe commercially advertised foods are healthy [16] and that commercially produced complementary food will make their children smart [17].

This research sought to establish the relationship between broad food systems and the nutritional status of children aged 6–24 months, and they were three research questions.

1. To assess the nutritional status of children between 6–24 months in Obunga slums in Kenya.
2. To determine the relationship between food price perceptions and the nutritional status of children between 6–24 months of age.
3. To determine the relationship between food and beverage marketing and the nutritional status of children between 6–24 months of age.

## Research methodology

### Study area

The Obunga slums are located in Kisumu County, on the Eastwards side; the coordinates are -004′44" N and 34,045′53E. It is in a total land area of 1.39 sq. km. It encompasses five smaller regions, Kasarani, Central 1, Central 2, Kamaokowa and Sega Sega. Obunga slums are next to the Kisumu Industrial area; it has emerged due to a shortage of affordable housing in Kisumu City.

### Study population

The study focused on children between 6–24 months residing in Obunga slums, plus their caregivers. The household was the unit of analysis, and respondents were the caregivers with children between 6–24 months.

Inclusion criteria included households with children aged 6–24 months residing in the Obunga slums.

Exclusion Criteria: Households with children aged 6–24 months residing in the Obunga slums had deformities and abnormalities (congenital disorders). As they are nutritionally vulnerable [18], The congenital disorders were established through observations, caregivers' reports and child records in the Mother and Child Booklet.

### Study design

A cross-sectional design was adopted, where data was collected once in March 2019 and analyzed. This design makes it easier to rapidly and effectively identify the relationship between the study's dependent and independent variables and to collect quantitative data (Ferderer, 2005). The design will enable the constitution of a hypothesis that can be subjected to analytical study. The merits behind cross-sectional design include; exposure and outcome being measured simultaneously, data being collected once and then analyzed, and it describes both absolute and relative risks.

### Sample size determination and the sampling procedure

#### Sample size determination

The sample size was determined according to Fisher et al., (1991) using the formula.

$$n=\frac{{Z}^{2}\left(\mathrm{pq}\right)}{{\mathrm{d}}^{2}}$$

Where:

n = represented the minimum sample size (for a population > 10,000) required

Z = the standard normal deviate at the required confidence level (set at 1.96 corresponding to 95%, Confidence level adopted for this study)

p= population proportion estimated to be stunted in Obunga. This now stands at 40.2% (Okeyo, 2015)

q = 1-pd= level of statistical significance set (5%)

Therefore, on substitution

$$\mathrm{n}=[{1.96}^{2}\times 0.402\times (1-0.402)]/{0.05}^{2}=369.40$$

However, since the targeted population was 274 eligible households, the final sample size (nf) was adjusted as follows:

$$\mathrm{nf}=\mathrm{n}\div \{1\div (\mathrm{n}/\mathrm{N})\}$$

Where:

 nf = desired sample size (when the target population is less than 10,000) 1.742

n= desired sample size (when the target population is greater than 10,000)

N= the desired sample size (target population)

$$\mathrm{nf}=274\div \{1+(274/369.40)\}=157.29$$

A non-response rate of 20% was added to cover the anticipated non-responses and fouled (spoilt) questionnaires [7].

$$157.29+(20/100) 157.29=188.748\approx 189$$

#### Sampling procedure

Listing was done to ascertain the actual numbers because Obunga slums have been due to the rapid migration in and out of the slums. Simple random sampling was then used to select 189 households from the 274 households listed in February 2019 as having children between 6-24 months. This was done in the following way.

1. The first step was to assign all the households with children between 6–24 months, numbers 1–274, having determined the population size of 274 and a sample size of 189.
2. Step 2 established a starting point by randomly opening a page and dropping a finger on the page with closed eyes.
3. In Step 3, four pieces of paper were written to choose the direction (up to down, down to up, left to right, and right to left). The pieces of paper were folded, shaken, and the direction from left to right was chosen.
4. In Step 4: The first unique 189 numbers were selected by reading from a table whose last three digits were between zero and 274. This was done because 274 is a 3-digit number.
5. Numbers were not repeated once chosen.
6. In Step 5, A new starting point was chosen; as we arrived at the end of the table, before meeting the target of 189 unique numbers, the direction was changed to up and down, and the target was achieved.

### Data collection instruments

A questionnaire was developed for this study and is supplementary file 1. The questionnaire had the following sections.

#### Questionnaire

An interviewer-administered questionnaire collected data on food price perceptions and Food & Beverage Marketing.

#### Anthropometric assessment form

An anthropometric data collection form was used to gather information on the children's height, weight and age.

### Data collection procedures

A. Questionnaire
  The researcher collected data through a face-to-face interview in the selected households using a questionnaire built into the mobile app-kobo-collect. Data was collected on food price perceptions and food & beverage marketing.
  - Food price perceptions: Data was collected on the consumption of the twelve food groups they included: cereals and grains; roots and tubers; legumes, seeds and nuts; milk and milk products; flesh meat and meat products; fish and other seafood; organ meat; eggs; Vitamin A rich fruits; other fruits; Vitamin A rich vegetables; Dark green leafy vegetables; Other Vegetables.
    Data collection was done according to the caregivers’ perception of the food price; Caregivers were requested to rate the food price from the twelve food groups [19] into either low, middle, or high.
  - Food and beverage marketing: Data was collected on exposure to media. This was checked by the frequency of reading newspapers, listening to the radio, watching television, and accessing social media. Data was also collected on promotional practices observed by mothers on commercially produced complementary foods since the child's birth, and if so, where they had seen or read the promotion. Finally, data were collected on the utilization of these foods, measured by caregivers reporting feeding their child any commercial food products before the interview day.
B. Anthropometric assessment
  This was measured through the recumbent length of each child. Each child’s length was measured twice to the nearest 0.1cm, and measurements were repeated when there was a deviation of >±0.5 cm. This was done through an infant/child length and height wooden measuring board by UNICEF (S0114530 Portable baby/child L-hgt mea.syst/SET-2). A child would be placed between the two slanting sides on their back. The head would gently be put against the top end, and the legs gently pushed downwards by the caregiver. The foot piece was slowly moved to the child until it pressed softly against the child’s soles, and the child’s feet were at right angles to the legs.
  Weight was then measured to the nearest 0.1g using SECA Model 881 digital scale (SECA GmbH, Hamburg, Germany). The children would have minimal clothing to avoid errors.
  The weighing scale was calibrated by placing a standard 20-kilogram weight on the scale every morning to ensure the scale could accurately measure 20 kg. If any error was seen, the scale was adjusted. The standard weight would be placed on the scale three consecutive times to ensure it has similar results three times, ascertaining its reliability. The anthropometric measures were done by taking two measurements of weight and two measurements of height; if the weight measure varied by plus or minus 0.1kg, it would be repeated. It would also be repeated if the height measure varied by plus or minus 0.1 cm.

### Pre-testing

The pre-testing was done on 19 respondents, who accounted for 10% [20] of the calculated sample. After which appropriate adjustments were made to the tool. The pre-testing was done in the Nyalenda slums, an informal settlement in Kisumu County similar to the Obunga slums. The results obtained helped to rework the questionnaire and standardize it.

### Validity and reliability

Content validity, which had to do with the instrument's format, including clarity of printing, size of type, adequacy of workspace, appropriateness of language and clarity of directions [21], was achieved by giving the instruments to the nutrition professionals to go through. Both face validity and content validity were ascertained.

Test re-test reliability was used to assess the consistency of a measure from one time to another. The time between one test and the other was one week. The correlation increases with decreasing time gaps, whereas decreasing time gaps result in a lesser correlation. This is due to the two observations' temporal correlation; the closer the time, the more similar the error-causing elements will be [21]. The validity of the weighing scale was ensured by placing a standard 20 kg measure on the weighing scale and calibrating it to ensure it read 20 kg. The reliability was confirmed by repeating this three times. Validity of the Height Board was done by placing a standard 1-m ruler on the height board and measures taken. This was repeated three times to ensure reliability.

### Study variables

A. Independent variables
  The Independent variables were
  - Food Price Perceptions: comprises data on the rating of food prices from the following food categories cereals and grains; roots and tubers; legumes, seeds and nuts; milk and milk products; flesh meat and meat products; fish and other seafood; organ meat; eggs; Vitamin A rich fruits; other fruits; Vitamin A rich vegetables; Dark green leafy vegetables; Other Vegetables [19]
  - Food and Beverage Marketing: This comprised data on access to media (television, radio, newspapers, and social media) and the promotional practices observed by the caregivers on commercially produced complementary foods and utilizing these foods.

The information solicited was used to facilitate the assessment of broad food systems that can influence the nutritional status of children living in Obunga slums between 6-24 months of age.

B. Dependent variable
  The nutritional status of the child was the dependent variable. This was treated as both a categorical variable and a continuous variable. The categorical variables were wasting, stunting, and underweight, which reflects WAZ, LAZ, and WLZ below -2 standard deviations, below the population median, and overweight, the WLZ above two standard deviations above the population median. To measure the length for age Z Scores (LAZ), the child's length and age were plotted against the WHO Length for age growth charts. To measure weight for length Z-scores (WLZ), the child's weight and age were plotted against the WHO weight for length growth charts. To measure weight for age Z-Scores (WAZ), the child's weight and age are plotted against the WHO weight for age growth charts.

### Data analysis

Data was imported from Kobo Collect to Microsoft Excel. Anthropometric data and information were entered into the ENA for SMART Software. Scores for height and nutritional status were generated based on WHO Child Growth Charts and Reference 2007 charts for children aged up to two years. Then all the data was imported into the Statistical Package for Social Sciences (SPSS) Version 25 (Illinois, Chicago). Descriptive statistics and Inferential Statistics were used to analyze data. Frequencies and proportions presented data analyzed through descriptive statistics through tables and text. Binary logistic regression was used to determine the relationship between food price perceptions, food and beverage marketing and the nutritional status of children between 6–24 months in Obunga slums. Crude Odds Ratio (COR) and Adjusted odds ratio (AOR) with Confidence Interval (C.I.) were then computed in binary logistic regression based on a 95% level of significance. To test the strength of the association between nutritional status, food price perception, and food and beverage marketing in the Obunga slums.

## Results

The study findings are presented under the following sub-headings per the study objectives: Nutritional status of children between 6–24 months and the relationship between food price perceptions and nutritional status, food and beverage marketing and nutritional status.

### Nutritional status of children between 6–24 months

The study had 189 children, and they all completed the study. 108 (57.1%) males and 81 (42.9%) females aged 6–24 months. The prevalence of wasting was 6 (3.2%), and girls were more wasted than boys at 4.9% and 1.9% per cent, respectively. The prevalence of overweight was 13 (6.9%), and girls were still more overweight than boys at (9.9%) and 4.6%, respectively. The prevalence of stunting was 27.0%, with boys more stunted than girls at 31.5% and 21%, respectively. The prevalence of underweight was at 7.4%, with boys more underweight at 9.3% and girls at 4.9%, as seen in Table 1.

**Table 1:** Distribution of children by nutritional status prevalence and sex *n* = 189

	**Gender**					
	**Female**		**Male**		**Total**	
	**N**	**(%)**	**N**	**(%)**	**N**	**(%)**
Wasted (< -2 z-score)	4	(4.9%)	2	(1.9%)	6	(3.2%)
Overweight (> 2 z-scores)	8	(9.9%)	5	(4.6%)	13	(6.9%)
Stunted (< -2 z-score)	17	(21%)	34	(31.5%)	51	(27.0%)
Underweight (< -2 z-score)	4	(4.9%)	10	(9.3%)	14	(7.4%)
Normal	48	(59.3%)	57	(52.8%)	105	(55.6%)

### Broad food systems and nutritional status

#### Food consumption patterns

The results show the proportion and percentage of children that consumed food from a particular food group the week before the data collection. Cereals and grains were the most consumed food by 98.9% of the children, while the least consumed was organ meats by 6.3% of the children, as seen in Table 2.

**Table 2:** Food consumption patterns

Characteristics	Frequency	Proportion (%)
**Cereals & Grains**		
Yes	187	98.9
**Roots & Tubers**		
Yes	122	64.6
**Legumes, Nuts & Seeds**		
Yes	114	60.3
**Milk & Milk Products**		
Yes	144	76.2
**Meat**		
Yes	60	31.7
**Fish**		
Yes	138	73.0
**Organ meat**		
Yes	12	6.3
**Eggs**		
Yes	109	57.7
**Vitamin-A rich fruits**		
Yes	90	47.6
**Other Fruits**		
Yes	137	72.5
**Vegetables**		
Yes	128	67.7

### Food prices perception

#### Food prices perceptions

The results show the food price perceptions of the caregivers on different food groups. The foods considered most expensive included organ meats, with 65.6% of the population finding them too expensive. In comparison, eggs were perceived to be low in price by 40.2% of the population. This is seen in Table 3 below.

**Table 3:** Food price perceptions

Food Item	Price Perceptions		
	**Low**	**Middle**	**High**
Cereals & Grains	13 (6.9%)	115 (60.8%)	61 (32.3%)
Roots & Tubers	5 (2.6%)	67 (35.4%)	117 (6.9%)
Legumes	15 (7.9%)	115 (60.8%)	59 (31.2%)
Milk	19 (10.1%)	146 (77.2%)	24 (12.7%)
Meat	6 (3.2%)	98 (51.9%)	85 (45.0%)
Fish	5 (2.6%)	97 (51.3%)	87 (46.0%)
Organ meat	4 (2.1%)	61 (32.3%)	124 (65.6%)
Eggs	76 (40.2%)	91 (48.1%)	22 (11.6%)
Vitamin A rich Fruits	39 (20.6%)	76 (40.2%)	74 (39.2%)
Other Fruits	32 (16.9%)	89 (47.1%)	68 (36.0%)
Vegetables	26 (13.8%)	62 (32.8%)	101 (53.4%)

#### Food prices perception associated with food consumption

The results show the association between a particular food group's perceived price and consumption in the previous week. The results showed that price increases of some foods decreased consumption of the foods. They include roots and tubers (O.R. = 0.45, C.I. = 0.25—0.28, *P* < 0.05), Legumes (O.R. = 0.56, C.I. = 0.34—0.95, *p* < 0.05), Organ meat (OR. = 0.29, C.I. = 0.11—0.77, *P* < 0.05) and Eggs (O.R. = 0.44, C.I. = 0.28—0.70, *p* < 0.05). The implication was that a 1 unit increase in the price of roots and tubers reduced intake of the roots and tubers by 0.45 times, consumption of legumes by 0.56 times, consumption of organ meat by 0.29 times, and consumption of eggs by 0.44 times. However, an increment in the price of vegetables significantly increased the consumption of vegetables (O.R = 0.43, C.I.—1.442- 3.43). This implied that a 1-unit increase in the price of vegetables increased consumption, as seen in Table 4.

**Table 4:** Food price perception of different foods associated with their food consumption

Characteristics	Significance	Crude O.R	Confidence intervals
Cereals & Grains	0.53	2.19	0.19—25.08
Roots & Tubers	0.01*	0.45	0.25—0.83
Legumes	0.03*	0.56	0.34—0.95
Milk	0.32	.696	0.34—1.41
Meat	0.15	.668	0.38—1.16
Fish	0.58	.844	0.47—1.53
Organ meat	0.01*	0.29	0.11—0.77
Eggs	0.001*	0.44	0.28—0.70
Vitamin A rich Fruits	0.75	.939	0.64—1.37
Other Fruits	0.63	.893	0.57—1.41
Vegetables	.000*	2.22	- 3.43

^*^ p < 0.05

### Relationship between food price perception and nutritional status

The results revealed that, while the price of other fruits increased, it significantly contributed to wasting at an (Adjusted O.R. = 10. 82, C.I. = 1.10—106.77, *P* < 0.05), and also contributed to underweight at an (Adjusted O.R. = 5.44, C.I. = 1.35—21.61, *P* < 0.05). The implication was that a 1 unit increment in the price of fruits contributed to an increase in wasting by 10.8 times and underweight by 5.4 times) as revealed in Table 5.

**Table 5:** Relationship between food price perception and nutritional status

Characteristics	Sig	Crude O.R	Confidence intervals	Sig	Adjusted OR	Confidence Intervals
**Wasting**						
Cereals & Grains	0.29	2.24	0.50—9.98	0.44	1.84	0.38—8.78
Roots & Tubers	1.00	0.00	0.00	1.00	119,272,130.1	0.00
Legumes	0.67	1.36	0.33—5.62	0.54	0.52	0.06—4.29
Milk	0.46	1.87	0.36—9.82	0.51	2.73	0.14—52.93
Meat	0.71	1.33	0.30—5.96	0.95	1.06	0.15—7.60
Fish	0.65	0.71	0.16—3.09	0.17	0.20	0.02—1.98
Organ meat	0.88	1.13	0.23—5.62	0.80	0.78	0.11—5.43
Eggs	0.66	1.32	0.40—4.37	0.65	1.51	0.26—8.81
Vitamin A rich Fruits	0.63	1.33	0.42—4.12	0.15	0.24	0.03—1.68
Other Fruits	0.11	3.46	0.75 -16.01	0.04*	10.82	1.10—106.8
Vegetables	0.72	1.25	0.37—4.17	0.97	1.03	0.20—5.20
**Stunting**						
Cereals & Grains	0.09	0.61	0.34—1.08	0.12	0.61	0.33—1.14
Roots & Tubers	0.15	1.59	0.84—2.00	0.42	1.34	0.66—2.75
Legumes	0.97	1.01	0.58—1.76	0.72	1.13	0.59—2.18
Milk	0.25	0.67	0.34—1.33	0.12	0.52	0.23—1.18
Meat	0.10	1.67	0.92- 3.04	0.06	1.92	0.96—3.82
Fish	0.74	0.90	0.50—1.63	0.69	0.87	0.46—1.67
Organ meat	0.26	1.45	0.76—2.78	0.50	1.29	0.62—2.66
Eggs	0.27	0.76	0.46—1.25	0.57	0.83	0.44—1.56
Vitamin A rich Fruits	0.44	1.19	0.77- 1.83	0.31	1.42	0.72—2.79
Other Fruits	0.95	1.02	0.64—1.61	0.62	0.84	0.41—1.71
Vegetables	0.39	1.22	0.77—1.94	0.22	1.42	0.81—2.49
**Underweight**						
Cereals & Grains	0.48	1.41	0.54—3.70	0.46	1.49	0.52—4.29
Roots & Tubers	0.72	1.21	0.43—3.45	0.67	1.31	0.37—4.59
Legumes	0.28	0.60	0.23—1.53	0.07	0.31	0.09—1.08
Milk	0.83	0.88	0.28—2.77	0.42	0.54	0.12—2.41
Meat	0.12	2.36	0.80—7.02	0.07	3.25	0.92—11.51
Fish	0.97	0.98	0.36—2.65	0.25	0.49	0.14—1.67
Organ meat	0.56	1.40	0.45—4.35	0.76	1.22	0.33—4.55
Eggs	0.21	1.67	0.75—3.72	0.18	2.19	0.70—6.81
Vitamin A-rich fruits	0.38	1.41	0.66—3.04	0.24	0.48	0.14—1.65
Other fruits	.042*	2.63	1.04—6.68	0.02*	5.44	1.37—21.61
Vegetables	0.35	1.51	0.64—3.54	0.45	1.51	0.52—4.38

^*^ p < 0.05

### Food and beverage marketing

#### Food and beverage marketing characteristics

The results showed that 28% of the population had listened to, read, or watched promotional practices on commercially produced complementary food. In comparison, only 6.9% fed their children commercially produced complementary food. On the other hand, more than ¾ % of the population had listened to, read, or watched promotional practices on commercially produced food products 76.7%, while 26.5% had fed their children these foods, as seen in Table 6 below.

**Table 6:** Food and beverage marketing characteristics

Characteristics	Frequency	Proportion (%)
**Frequency of reading Food-related adverts in Newspapers**		
Never	131	69.3
Rarely	56	29.6
Monthly	1	.5
Weekly	1	.5
Daily	0	0
**Frequency of watching food-related adverts on television**		
Never	38	20.1
Rarely	76	40.2
Monthly	0	0
Weekly	32	16.9
Daily	43	22.8
**Frequency of listening to food-related adverts on radio**		
Never	35	18.5
Rarely	58	30.7
Monthly	3	1.6
Weekly	27	14.3
Daily	66	34.9
**Frequency of listening, reading, or watching food-related content/adverts on social media**		
Never	153	81.0
Rarely	25	13.2
Monthly	0	0
Weekly	3	1.6
Daily	8	4.2
**Marketing on Complementary food**		
Yes	53	28.0
**Marketing on other Commercial food**		
Yes	145	76.7
**Child consumption of commercially produced complementary food products**		
Yes	13	6.9
**Child consumption of commercially produced food products**		
Yes	50	26.5

#### Food and beverage marketing associated with nutritional status

The results reveal that children fed on commercially produced complementary food products and commercially produced food significantly contributed to an increase in wasting at an (Adjusted O.R. = 7.82, C.I. = 1.29—47.46, *p* < 0.05, and adjusted O.R. = 5.96, C.I. = 1.06—33.60, *p* < 0.05) respectively. The implication was a 1 unit increase in consumption of commercially produced complementary food products and commercially produced foods, which contributed to the rise in wasting by 7.8 times and 6 times, respectively. Regarding stunting, the results revealed that listening/reading or watching promotional practices on commercial food products contributed to a decrease in stunting at (Crude O.R. = 0.49, C.I. = 0.24—0.998) *p* < 0.05. This suggests that a 1 unit increase in reading/listening or watching promotional practices on commercially produced food reduced stunting by 0.5 times. In the adjusted model, promotional practices also contributed to a decrease in stunting, Where watching food-related adverts on television reduced stunting at an (Adjusted O.R. = 0.38 C.I. = 0.146- 0.10) and watching marketing on commercial foods reduced stunting at an (Adjusted O.R. = 0.21, C.I. = 0.07—0.61). However, watching television increased stunting 9 times (Adjusted O.R. = 9.30, C.I. = 2.31—37.40). Regarding being underweight, the results revealed that watching food-related adverts on television reduced the prevalence of being underweight by 18 times (Adjusted O.R. = 18.68 and at C.I. = 1.22—286.89), as seen in Table 7.

**Table 7:** Food and beverage marketing associated with nutritional status

Characteristics	Sig	Crude O. R	Confidence intervals	Sig	Adjusted OR	Confidence intervals
**Wasting**						
**Marketing channel**						
Newspaper	0.89	1.13	0.20—6.37	0.63	1.82	0.16—20.79
Television	1.00	0.00	0.00	1.00	0.00	0.00
Radio	1.00	0.00	0.00	1.00	0.00	0.00
Social Media	0.88	0.85	0.10—7.47	0.41	0.36	0.03—3.98
**Marketing on Complementary food**	0.24	2.66	0.52—13.62	0.44	2.36	0.27—21.07
**Marketing on other Commercial food**	0.70	1.54	0.18—13.51	0.71	0.56	0.03 – 12.0
Child consumption of commercially produced complementary food products	0.03*	7.82	1.29—47.46	0.57	2.00	0.18—22.11
Child consumption of commercially produced food products	0.04*	5.96	1.06—33.60	0.10	6.40	0.71—57.73
**Stunting**						
**Marketing Channel**						
Newspaper	0.82	0.92	0.46—1.86	0.05*	0.38	0.15—1.001
Television	0.09	2.26	0.89—5.79	.002*	9.30	2.31—37.40
Radio	0.85	1.08	0.47—2.50	0.80	0.86	0.28—2.68
Social Media	0.77	0.88	0.38—2.02	0.51	1.38	0.53—3.61
**Marketing on Complementary food**	.057	0.46	0.21—1.023	0.25	0.59	0.24—1.46
**Marketing on other Commercial food**	.049*	0.49	0.237—0.998	.004*	0.21	0.07—0.61
Child consumption of commercially produced complementary food products	1.00	0.00	0.00	1.00	0.00	0.00
Child consumption of commercially produced food products	0.36	0.70	0.33—1.50	0.58	0.78	0.32—1.90
**Underweight**						
**Marketing Channel**						
Newspaper	0.86	0.90	0.27—2.99	0.33	0.45	0.09—2.24
Television	0.24	3.49	0.44—27.51	.036*	18.68	1.22—286.89
Radio	0.77	0.82	0.22—3.11	0.31	0.43	0.08—2.20
Social Media	0.26	0.31	0.04—2.43	0.29	0.31	0.04—2.71
**Marketing on Complementary food**	0.57	0.68	0.18—2.55	0.39	0.47	0.08—2.67
**Marketing on other Commercial food**	0.26	0.52	0.16—1.63	0.07	0.21	0.04—1.14
Child consumption of commercially produced complementary food products	0.27	2.49	0.49—12.51	.417	2.48	0.28—22.28
Child consumption of commercially produced food products	0.16	2.23	0.73—6.79	.129	2.92	0.73—11.63

^*^ *p* < 0.05

## Discussion

### The nutritional status of children

Globally 23% of children are stunted, representing chronic malnutrition, while wasting is at 7.5%, and being overweight is also steadily rising [1, 2]. The results from this study reveal that the prevalence of stunting and being overweight in Obunga slums was higher than at the national and county [2]. However, compared to other studies done in Obunga slums [7], A sentinel survey by Kisumu Medical and Education Trust, 2011, and other slums in Nairobi [5, 6]. Stunting has reduced drastically. However, the difference might be because the other studies focused on children under five years of age, while in this study, the focus was on children between six and twenty-four months. While undernutrition is expected among the urban poor, over-nutrition is rising; this has also been reflected in other urban slums [22, 23]. However, the prevalence of wasting and being underweight was lower than the national prevalence but higher than the county prevalence [2]. However, the prevalence of stunting and underweight was in the same range as the other slum areas in Kenya [6, 23]. The study clearly showed that children between 6–24 months in urban slums contribute to the national burden of malnutrition.

### Relationship between the food price perception, food & beverage marketing and the nutritional status

#### Food price

Food price is an essential determinant in food purchases [12]. Rising food prices decrease a household's purchasing ability [24]. To cope with the increasing food prices, there is usually a reduction in both the household's energy intake and dietary diversity [14]. This study showed that an increase in food prices was significantly associated with a reduction in the consumption of roots and tuber foods, legumes, organ meat and eggs, but the consumption of vegetables increased. The increase in vegetable consumption was contradictory to a study by [25], who found that rising food prices would reduce the consumption of vegetables but agreed with [24, 26, 27], who showed that an increase in food prices reduced the consumption of animal-source foods. The reduction in food quality has been identified as a coping mechanism; among vulnerable populations [14, 28, 29], whereas food prices reduce diet quality and quantity [14, 28]. The increase in vegetable consumption was possibly because, In Kisumu County, where Obunga is located. The staple cereal is maize prepared as ugali (a stiff porridge commonly consumed with a meat stew, fish or vegetables) or as githeri (a mixture of maize and beans). The increased food price led to reduced consumption of legumes, roots and tubers and animal-source foods. It is then assumed that vegetables became the main accompaniment for ugali.

This study also found that increasing the price of fruits was significantly associated with wasting and being underweight. High food prices have been associated with undernutrition [28, 30]. In Bangladesh, high expenditure on rice, compared to non-rice items, increased the odds of child stunting. At the same time, a reduction was seen in households where rice expenditure was low [24]. High spending on rice reduced the money available to purchase animal-source food, vegetables, fruits and oils, reducing food quality [24]. In Indonesia, households that spent a large percentage of their income on animal-source foods had a lower prevalence of stunting [26]. Food from animal sources is a high-quality protein, rich in micronutrients Vitamin B-12, Iron, Riboflavin, Vitamin A, phosphorous, calcium and zinc, and essential fatty acids [24, 31]. Intake of these foods by children reduces stunting, as they boost growth, micronutrient status & cognitive development in children [31]. As food prices continue increasing, the meal portion sizes reduce, and the frequency of meals decreases as well; this reduces the staples consumed, leading to macro-nutrient deficiencies and increasing the risk of acute malnutrition amongst children [14]. It is fascinating to note that rising fruit and vegetable prices have also been linked to children's body mass indices that are higher [32], while subsidies on fruits & vegetables were shown to improve children's & adolescents' weight outcomes [33].

#### Food and beverage marketing

Food and Beverage marketing promotes particular brands and food categories [12], with children being intensively targeted by food marketing and advertisements [15]. This study shows that caregivers' exposure to commercially produced food products was high at over 75% compared to exposure to commercially produced complementary food products, representing 28% of the population. The trend was similar to other studies that have been done both on commercially produced food products and commercially produced complementary food products [16, 34]. Exposure to food commercials has increased Obesity among children, adolescents and adults [35]. In this study, however, stunting was also decreased amongst children whose caregivers listened, read or watched promotional practices on commercial foods. However, watching television food-related adverts increased the underweight prevalence in the study. This was in agreement with [36], who found that watching television was associated with poor dietary quality. Children consumed more unhealthy foods, including sugar-sweetened beverages, chips and crackers, leading to poor weight outcomes. This study showed that feeding children commercially produced food complementary foods and commercially produced foods contributed to an increase in wasting. This might be because most of the commercial snacks and food products are usually high in sugar, sodium, fat and are usually of low nutritional quality [16, 17, 34, 37, 38].

## Conclusion

In Obunga slums, the prevalence of stunting, an indicator of chronic malnutrition, was 27%, higher than the national average. The prevalence of overweight 6.9% was also greater than the national average, while underweight 7.4% and wasting prevalence of 3.2% were lower than the national average. We realize that food prices and food and beverage marketing impact the nutritional status of children between 6–24 months. Thus, the need for a longitudinal study would better understand the long-term effect of food prices and food and beverage marketing on the nutritional status of children 6–24 months in Obunga slums, Kenya.

### Supplementary Information

**Additional file 1.**

## Data Availability

Data is available upon request from the corresponding author.

## Abbreviations

AOR: Adjusted Odds Ratio
C.I.: Confidence Intervals
KDHS: Kenya Demographic Health Survey
LAZ: Length for Age Z-score
OR: Odds Ratio
UNICEF: United Nations Children's Fund
WAZ: Weight for Age Z-Scores
WHO: World Health Organization
WLZ: Weight for Length Z-Score
Z-Score: The number of standard deviations from the mean of the Datapoint
