# Supplementary material for: Food price perception, food and beverage marketing and the nutritional status of children 6–24 months in Obunga slums, Kisumu Kenya: a cross-sectional study
Source: BMC Nutr. 2023 Oct 6;9:114. doi: 10.1186/s40795-023-00772-3 (PMC10559538; doi:10.1186/s40795-023-00772-3)
Supplement: Supplementary file 1 — Additional file 1. [file 40795_2023_772_MOESM1_ESM.docx]

**Data Collection Instruments.**

**Questionnaire no: _______________ Date: ______________ Village__________**

**Section A: Anthropometric Assessment Form**

Height: Measure 1____________________ Measure 2____________________

Weight: Measure 1___________________ Measure 2___________________

Child’s Gender: _________________ Child’s Age in Months: ____________________

**Section B: BROAD FOOD SYSTEMS**

**Food Consumption Patterns**

| 1. | Has the child consumed foods from the following food groups in the past week? | | |
| --- | --- | --- | --- |
| a. | **Cereals and Grains** (maize and maize products, rice, spaghetti, noodles, indomie, wheat, and wheat products) | Yes  No | 1  2 |
| b. | **Roots and Tubers** (Irish potatoes, sweet potatoes, green bananas, arrowroot, and cassava) | Yes  No | 1  2 |
| c. | **Legumes/Nuts/seeds**(beans, ndengu, Kunde (cowpeas), njahe (black beans), mbaazi, lentils | Yes  No | 1  2 |
| d. | **Milk and Milk products** (milk, yoghurt, maziwa mala, cheese, butter, cream) | Yes  No | 1  2 |
| e. | **Flesh meat and meat products** (beef, goat, pork, mutton, chicken, fish) | Yes  No | 1  2 |
| f. | **Fish and other seafood** (Omena, tilapia, fulu, Nile perch) | Yes  No | 1  2 |
| g. | **Organ meat** (matumbo, heart, liver, kidneys) | Yes  No | 1  2 |
| h. | **Eggs** (chicken, duck, quail) | Yes  No | 1  2 |
| i. | **Vitamin A rich fruits** (ripe mango, ripe papaya) | Yes  No | 1  2 |
| j. | **Other fruits** (oranges, bananas, melons, pineapples, guava) | Yes  No | 1  2 |
| k. | **Vitamin A rich Vegetables & Dark Green Leafy Vegetables** (pumpkin, carrots, spinach, tomatoes, kales, spider plant, amaranth, jute mallow, black nightshade, cassava leaves) | Yes  No | 1  2 |
| l. | **Other vegetables** (tomatoes, onions, cabbage, eggplant) | Yes  No | 1  2 |

**Food Price Perceptions**

| **2.** | **Generally, how would you rate food prices from the following categories?** | | |
| --- | --- | --- | --- |
| a. | **Cereals and Grains** (maize and maize products, rice, spaghetti, noodles, indomie, wheat & wheat products) | Low  Very Low  Middle  High  Very High | 1  2  3  4  5 |
| b. | **Roots and Tubers** (Irish potatoes, sweet potatoes, green bananas, arrowroot, and cassava) | Low  Very Low  Middle  High  Very High | 1  2  3  4  5 |
| c. | **Legumes/Nuts/seeds**(beans, ndengu, Kunde (cowpeas), njahe (black beans), mbaazi, lentils | Low  Very Low  Middle  High  Very High | 1  2  3  4  5 |
| d. | **Milk and Milk products** (milk, yoghurt, maziwa mala, cheese, butter, cream) | Low  Very Low  Middle  High  Very High | 1  2  3  4  5 |
| e. | **Flesh meat and meat products** (beef, goat, pork, mutton, chicken, fish) | Low  Very Low  Middle  High  Very High | 1  2  3  4  5 |
| f. | **Fish and other seafood** (Omena, tilapia, fulu, Nile perch) | Low  Very Low  Middle  High  Very High | 1  2  3  4  5 |
| g. | **Organ meat** (matumbo, heart, liver, kidneys) | Low  Very Low  Middle  High  Very High | 1  2  3  4  5 |
| h. | **Eggs** (chicken, duck, quail) | Low  Very Low  Middle  High  Very High | 1  2  3  4  5 |
| i. | **Vitamin A rich fruits** (ripe mango, ripe papaya) | Low  Very Low  Middle  High  Very High | 1  2  3  4  5 |
| j. | **Other fruits** (oranges, bananas, melons, pineapples, guava) | Low  Very Low  Middle  High  Very High | 1  2  3  4  5 |
| k. | **Vitamin A rich Vegetables and Dark Green Leafy Vegetables** (pumpkin, carrots, spinach, tomatoes, kales, spider plant, amaranth, jute mallow, black nightshade, cassava leaves) | Low  Very Low  Middle  High  Very High | 1  2  3  4  5 |
| l. | **Other vegetables** (tomatoes, onions, cabbage, eggplant) | Low  Very Low  Middle  High  Very High | 1  2  3  4  5 |

**Food and Beverage Marketing**

| **No** | **Question** | **Choices** | **Response** |
| --- | --- | --- | --- |
| 1. | How often do you read food-related articles in newspapers? | Never  Rarely  Monthly  Weekly  Daily | 1  2  3  4  5 |
| 2. | How often do you watch food-related adverts or content on television? | Never  Rarely  Monthly  Weekly  Daily | 1  2  3  4  5 |
| 3. | How often do you listen to food-related content/adverts on the radio? | Never  Rarely  Monthly  Weekly  Daily | 1  2  3  4  5 |
| 4. | How often do you listen to, read, or watch food-related content/adverts on social media (Facebook, Instagram, WhatsApp, Twitter) | Never  Rarely  Monthly  Weekly  Daily | 1  2  3  4  5 |
| 5. | Have you listened to, read, or watched any promotional practices on commercially produced complementary food? | Yes  No | 1  2 |
| 6. | Have you listened to, read, or watched any promotional practices on commercially produced food products? | Yes  No | 1  2 |
| 7. | Did you feed your child any of the commercially produced complementary food yesterday? | Yes  No | 1  2 |
| 8. | Did you feed your child any of the commercially produced food products yesterday? | Yes  No | 1  2 |
